# Shall Children Be Insured?

**Published:** 1891-04-11

**Authors:** 


// The Hospital

A Weekly Journal of

Science, Medicine, Nursing, and Philanthropy.

*For Hospitals, Asylums, Infirmaries, Dispensaries, Medical Practitioners, Students, Nurses, and the Charitable Public.*

Vol. X.—No. 237.] [April 11, 1891.

### Shall Children be Insured?

The immediate occasion of this article is a controversy which has been lately carried on by Captain Pembroke Marshall and the Rev. Benjamin Waugh, of the Society for the Prevention of Cruelty to Children. A mere "hash" of this controversy is not to our mind, and would probably defeat the object we have in view. Certain facts brought forward by the controversialists may help our argument; and those we shall therefore use. But our desire is to approach the question at the head of the article from an independent standpoint.

The persons most vitally interested in Child Life Assurance are children. Children who do not even know that they are assured; and who could not form an opinion on the subject if they did know. Children, who, though they cannot think, can feel acutely; and who can die of neglect, or of starvation, or of poison, though they cannot reason about such things. Although children are so vitally interested in child assurance, they cannot by any possibility receive any benefit from it. They are not insured in order that their health may be promoted or their lives saved. That is not the object at all. They are insured in order that if they die money may pass into the pockets of their parents or guardians. Moreover, they can, when living, render no services to their parents at their tender age; they can, however, give those parents great trouble in their nursing and rearing; and they can also put them to great expense.

Now let any man of fifty imagine himself to be insured for ten thousand pounds; he has been overtaken by paralysis; he is helpless and querulous. Because he can no longer work the income from his business has ceased. His family therefore are in straits. They must keep up his insurance premiums, otherwise the considerable annual sums that have already been paid will be lost, and the prospective ten thousand pounds for which the life is insured will all be sacrificed. But now that the father's business income is gone, the means of bare livelihood are reduced to a minimum, and the payment of the annual insurance premiums is an utter impossibility. If the paralysed man would but die, ten thousand pounds would come in from the insurance office, which at four per cent. would yield an income of four hundred a-year. Not only so, but the annual premiums would no longer need to be paid. Moreover, the paralysed, querulous, and exacting father would cease to be a burden on an overwrought wife, or on under-paid sons and daughters. To such a household as is here supposed, the continued life of the assured means poverty, loss of sleep, injury to health, and every kind of harassment and worry. His death would be the beginning of competence, the lessening of expenditure, and the removal of a cause of incessant anxiety and wearing toil. In such a case it is not possible for ordinary human nature to avoid looking forward to the death of the assured, even though he be a husband and father, as to an occasion which will be the dawn of happiness and the end of care.

All that we have supposed in the case of an educated family of the middle class applies with the fullest force to an insured child of the poorer class. The child can bring no grist to the mill at its present tender age; it requires a great deal of nursing; if it be ill it disturbs the sleep of the father and mother and the other children with its fractious cries; it needs nourishment of a specially expensive kind; it wears out the mother's feeble strength, and diminishes the father's scanty wages. The death of the child would mean immediate relief from all these daily and nightly harassments, the diminution of expense, and a relatively large sum of ready money in the parents' pocket. Just as in the case of the paralysed father, it was humanly impossible to avoid looking upon his death with satisfaction, so in the case of the profitless and sickly infant, it is humanly impossible for the father and the mother of the lower social grade to avoid the feeling that death would be an advantage both to the child and to themselves.

Now our contention is that no infant ought to be placed in this unhappy position; and no parents ought to be subjected to such a convergence of temptations. As the law stands, the children of the rich cannot be insured at all. This is a fact full of striking significance: for the law which debars the rich from insuring their children was made by the rich. When the subject of child insurance was first under legislative consideration, it was held by legislators that such insurance was not permissible. The reason, of course, why this conclusion was arrived at, was that children could not be insured for their own benefit, but only for the benefit of somebody else; and that as the insured child would be in the position of a helpless person, living under the sole control of some one who might have no interest in his life but great interest in his death, to permit children under these circumstances to be insured was to offer a premium to child murderers. That was an entirely rational view of the situation; and as that view was almost universally held by legislators, it prevailed, and a statutory enactment was made that no child life should be insurable under a certain age.

But within a comparatively recent period a change was effected in the law, and it was made solely in the interests of the working classes. The children of working men can now be insured; but those of the better educated and more prosperous classes cannot. The intention of the legislature was good when this new enactment was made; but the result has been shocking and horrible. We purposely avoid entering into Mr. Benjamin Waugh's statistical details and into Captain Marshall's statistical refutations. The bare and simple truth is that child murder, as the result of child insurance, prevails in England to a very large extent. This is proved in a thousand unquestionable ways—by judges and policemen, by doctors and relieving officers, by coroners and enquiry agents. We need no more proof than that which has been furnished already by almost every large town and county in Great Britain. The proofs, unhappily, are a hundred times too many and too dreadful. In our judgment no candid or fair enquirer would think of disputing them. As a plain matter of fact, child murder prevails in this country, and it prevails most where children's lives are most insured.

Ought we then to put an end by statute to the system of child insurance altogether? We ought, if we desire to avoid complicity with murder, and with the kind of murder of which Herod was guilty —the murder of helpless innocents. Insurance offices themselves ought to be the very first to haul down the black flag of the murderous pirate, and to shake themselves free from all suspicion of such a horrible warfare against child life as has been carried on by their countenance and help. But how can the poor provide for the burial of their children in case of death except by some such means as certain insurance companies provide? This is, of course, the crucial question from the point of view of the public and the well-doing poor. It is usually supposed to be a difficult question; but the difficulty is of the most trifling kind compared with many of the difficulties which have been taken in hand and overcome by the people of this country since their emergence from the savage state.

Two methods of burying the dead children of the poor suggest themselves here, and others might be evolved by patient thought. The first is that of voluntary aid; the second is that of State provision. We have voluntary hospitals and other charitable institutions for the living: what is to prevent us from instituting voluntary charitable institutions for the decent burial of the dead children of the poor? With regard to State provision, it is already made of a certain kind. But for those honourable poor who object to pauper funerals, there is no reason whatever why a system of State loans should not be instituted, loans which should bear interest, and be repayable by such convenient instalments as the borrower might be able to afford. There are difficulties in both these plans, suggests the critic. Quite so! And there are disgraces connected with child murder, and with the impetus given to it by State countenance, which a proud and honourable nation like our own ought to cast from it as a man throws from him a loathsome serpent which he has caught in the very act of striking at his life.

